# Description of larval morphology and phylogenetic relationships of *Heterotemna tenuicornis* (Silphidae)

**DOI:** 10.1038/s41598-021-94744-x

**Published:** 2021-08-20

**Authors:** Karolina Mahlerová, Pavel Jakubec, Martin Novák, Jan Růžička

**Affiliations:** Faculty of Environmental Sciences, Czech University of Life Sciences Prague, Kamýcká 129, 165 00 Prague, Suchdol, Czech Republic

**Keywords:** Entomology, Phylogenetics, Biodiversity

## Abstract

Providing clear and detailed morphological descriptions of endemic species in limited areas enables new knowledge of their biology and ecology to be obtained through citizen science. This information can be further used for their protection. Our study presents the first morphological description of the larvae of all three instars of *Heterotemna tenuicornis* (Brullé, 1836), an endemic species of the Canary Islands that, together with *H. britoi* García & Pérez, 1996 and *H. figurata* (Brullé, 1839), belongs to the peculiar genus *Heterotemna* Wollaston, 1864. Furthermore, we present the first sequences of two mitochondrial genes (COI, 16S) obtained from larval specimens, and cross reference them with sequences from an adult specimen. Phylogenetic analysis of molecular data placed the genus *Heterotemna* within the genus *Silpha* Linnaeus, 1758, suggesting paraphyly of *Silpha*. In our study, we underline the importance of using a combination of morphological description and molecular data, that can be used for barcoding developmental stages which could not otherwise be definitely associated.

## Introduction

Protection of species requires the combination of many scientific disciplines, but the first and foremost problem often faced by conservationists is the accurate determination of the focal species^1^. Currently two main approaches to identification are recognized; molecular (via DNA barcoding) and morphological. The morphological approach may seem obsolete since the techniques have improved rapidly only in the last two decades, but it retains some advantages over DNA barcoding^2–4^. One of the most important of these, is that it does not require harming/killing the animal, which is crucial especially in the case of smaller and more fragile organisms such as insects. Furthermore, morphological identification empowers citizen scientists (non-professionals, enthusiasts, and even the general public) with the ability to recognize the species and help gather relevant information about its ecology, biology and distribution^5–7^.

Beetles are one of the foremost examples of how taxonomy can become polarized in terms of research interest. While adult beetles seem to have been in the spotlight of taxonomy for the last few 100 years, the larvae and other developmental stages have been far less studied despite the fact that many species spend the majority of their life in the larval stage, making these stages critical for conservation efforts (e.g.^8–10^). Knowledge of larval morphology is often very limited, which leads to a lack of information on their biology and ecology^11,12^. In terms of insects, crucial data about species are usually only collected when a species becomes endangered and is fortunate enough to attract some conservation attention, but this can often be too late. In order to involve the public in a new form of citizen science we should supply them with means of identification, so people can help collect data before they are actually needed to mitigate a potential crisis^6,7^.

In this article, we focus on *Heterotemna tenuicornis* (Brullé, 1836) (Coleoptera: Silphidae: Silphinae), one of three species of the genus *Heterotemna* Wollaston, 1864, endemic to the Canary Islands. The Canary Islands were formed of seven major islands of volcanic origin, creating approximately 500 km chain of islands available for colonisation for the past 15 – 20 million years^13–15^. The archipelago belongs to Macaronesia, which consists of five archipelagos located in the Atlantic Ocean (the Canary Islands, the Azores, Selvagens, Madeira and Cape Verde), the closest distance to the mainland is 96 km (North Africa, Morocco)^16,17^. The largest island of the Canary Islands is Tenerife, which was formed from two to possibly three isolated proto-islands joined together by volcanic eruption 1.9–0.2 million years ago^18^. The original proto-islands had served as a refuge for species that subsequently colonised the newly formed island^19,20^. Unique ecosystems such as *Euphorbia* scrubs, laurel forest (laurisilva), pine forests, and alpine scrub^21^ together with relatively recent volcanic activity and diverse landscapes created suitable conditions for Tenerife’s high endemism and unique composition of habitats^21–23^.

The subfamily Silphinae consists of 18 genera or subgenera^24^ and *Heterotemna* Wollaston, 1864 is the last genus of Silphinae lacking formal larval description and a clear phylogenetic placement within the subfamily. Only a single photograph of the dorsal side of a probably 3rd instar larva has been published so far, but without any further comment^25^. Relationships and taxonomic position of genera inside the internal group of the subfamily Silphinae^26^ were established based on morphological resemblances of adults, and they are not generally agreed upon. For example, the genera *Phosphuga* Leach, 1817 and *Ablattaria* Reitter, 1885 are often considered to be subgenera of the genus *Silpha*^24,27^. Detailed study coupled with molecular phylogenetic analysis would contribute towards our understanding of the evolutionary relationships within the subfamily Silphinae.

All three recognized *Heterotemna* species are considered to be forest inhabitants. However, *H. britoi* García & Pérez, 1996 and *H. figurata* (Brullé, 1839) can be also found in more open environments. *Heterotemna tenuicornis* is a common species that can be found in the interior of the laurel forest in the Teno and Anaga regions (Fig. 2b) under logs and in the litter throughout the year^25^. Based on its limited geographical range, confined only to Tenerife and La Palma, the whole genus *Heterotemna* could face several challenges. Until recently, *Heterotemna* were the only Silphidae known to inhabit the Canary Islands. However, the closely related *Silpha puncticollis* Lucas, 1846 was reported from Tenerife in 2010, suggesting its recent introduction; it has formed a viable population in La Laguna city^28^, and could pose a threat to the native species. Furthermore, climatic change is a serious threat to species with limited range and confined to small islands, as they have very limited options of evasion of unsuitable conditions. Knowledge of larval morphology is thus crucial for making the species available for citizen science-based studies and consequently for conservation measures.

The aims of this study are to produce the first morphological description of all instars of larvae of *H. tenuicornis*, and to investigate the phylogenetic placement of the genus at the subfamily level, and its association with the genus *Silpha*, based on molecular data (16S and COI).

## Results

**Figure 1:**
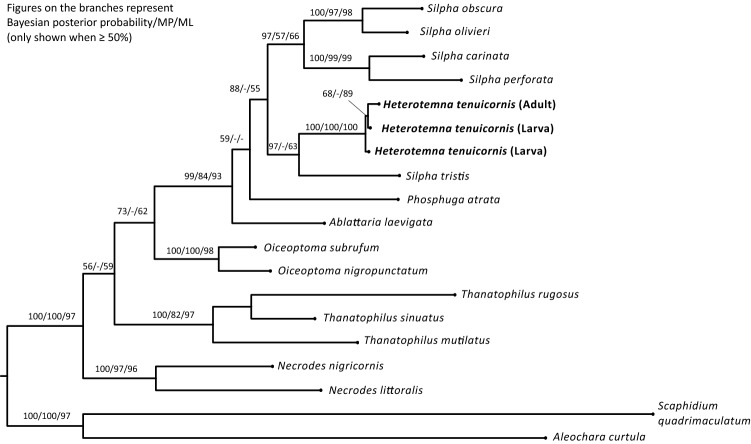
Phylogenetic tree based on Bayesian analysis. Numbers above branches show the posterior probability and bootstrap values (BI)/maximal parsimony (PAUP)/Maximum likelihood (MEGA). *Scaphidium quadrimaculatum* Olivier, 1790 and *Aleochara curtula* (Goeze, 1777) (both Staphylinidae) were selected as outgroups.

In total 48 larval specimens of *H. tenuicornis* were obtained and analysed. We identified 30 larvae of the first instar, 14 of the second instar and 4 of the third instar. Two larvae and one adult specimen of *H. tenuicornis* were used for molecular phylogenetic placement of the genus within the subfamily Silphinae. The phylogenetic tree was obtained using Bayesian analysis from the concatenated partial 16S (434 bp) and COI (609 bp) sequences (Fig. 1).

### Species identification based on genetic distances

The calculated p-distances between concatenated sequences of 16S and COI of larval and adult specimens of *H. tenuicornis* were between 0.0029 and 0.0078 (the mean calculated p-distance within *Heterotemna* specimens was 0.01). Conversely, the distance between different species of *Silpha* was shown to be higher (mean calculated p-distance within the *Silpha* species was 0.08), thus the larval specimens were confirmed as belonging to the same species as the adult specimen, *H. tenuicornis* (SM1).

### Phylogenetic analyses

The Bayesian analysis (posterior probability 99), maximum parsimony bootstrap (84) and maximum likelihood bootstrap (93) strongly supported a clade of the genera *Silpha*, *Heterotemna*, *Ablattaria* and *Phosphuga*, suggesting close relationships of these genera with *Heterotemna* inside the genus *Silpha*, which makes the genus *Silpha* paraphyletic. The position of *H. tenuicornis* as a sister lineage to *S. tristis* Illiger, 1798 was strongly supported by the Bayesian analysis (97) but not strongly supported by the other analyses. The results confirmed the monophyly of the genera *Thanatophilus* Leach, 1815, *Necrodes* Leach, 1815, and *Oiceoptoma* Leach, 1815 within the subfamily Silphinae (Fig. 1).

### Morphometry

**Figure 2:**
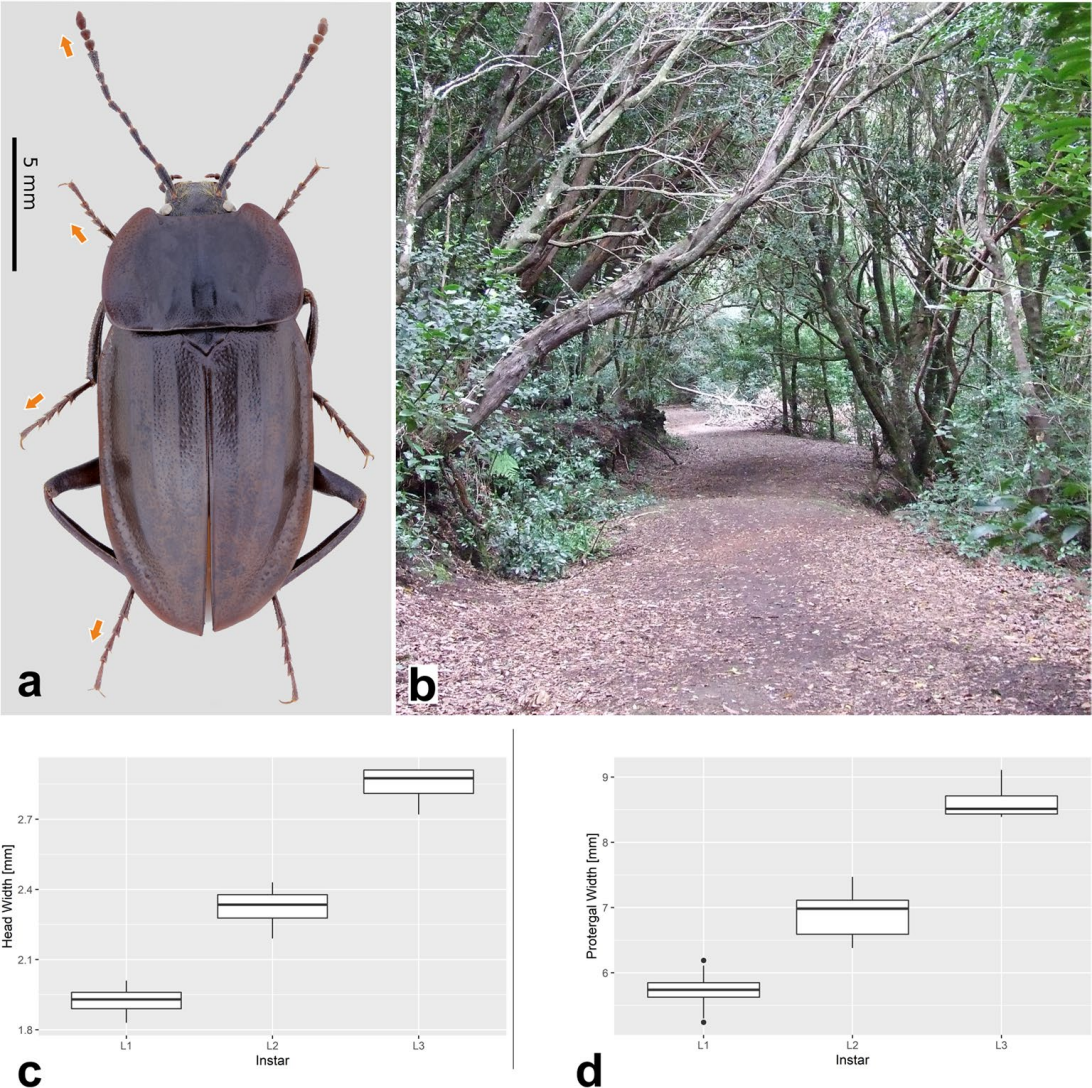
*Heterotemna tenuicornis* (Brullé, 1836): (**a**) female adult, dorsal view, arrows indicate distinctly elongated appendages; (**b**) Anaga Mts., detailed habitat with laurel forest. Boxplots of measurements of all three larval instars: (**c**) head width; (**d**) protergum width.

The two commonly used measurements for instar identification, head width and width of protergum , are applicable in the case of *H. tenuicornis* (Fig. 2c, d) as these two measurements do not overlap between the instars and show significant differences. More specifically, the following measurements were very different between instars; head width (F statistic = 231 on 2, df = 45, *p* value < 2.2e−16), protergal width (F statistic = 4.109 on 2, df = 45, *p* value < 2.2e−16). Significant difference was also observed in the length of the first segment of the maxillary palpus (F statistic = 9.181 on 2, df = 44 , *p* value < 0.0004653), all three antennomeres (AI F statistic = 112.3 on 2, df = 45, *p* value < 2.2e−16, AII F statistic = 143.2 on 2, df = 45, *p* value < 2.2e−16, AIII F statistic = 69.19 on 2, df = 45, *p* value 1.868e−14) and both urogomphal segments (UI F statistic = 95.25 on 2, df = 45 , *p* value < 2.2e−16, UII F statistic = 4.109 on 2, df = 45, *p* value 0.02296). In the case of the urogomphal segments, we observed isometric growth in the first segment but not in the second segment (SM4).

### Larval morphology

#### Diagnosis

**Figure 3:**
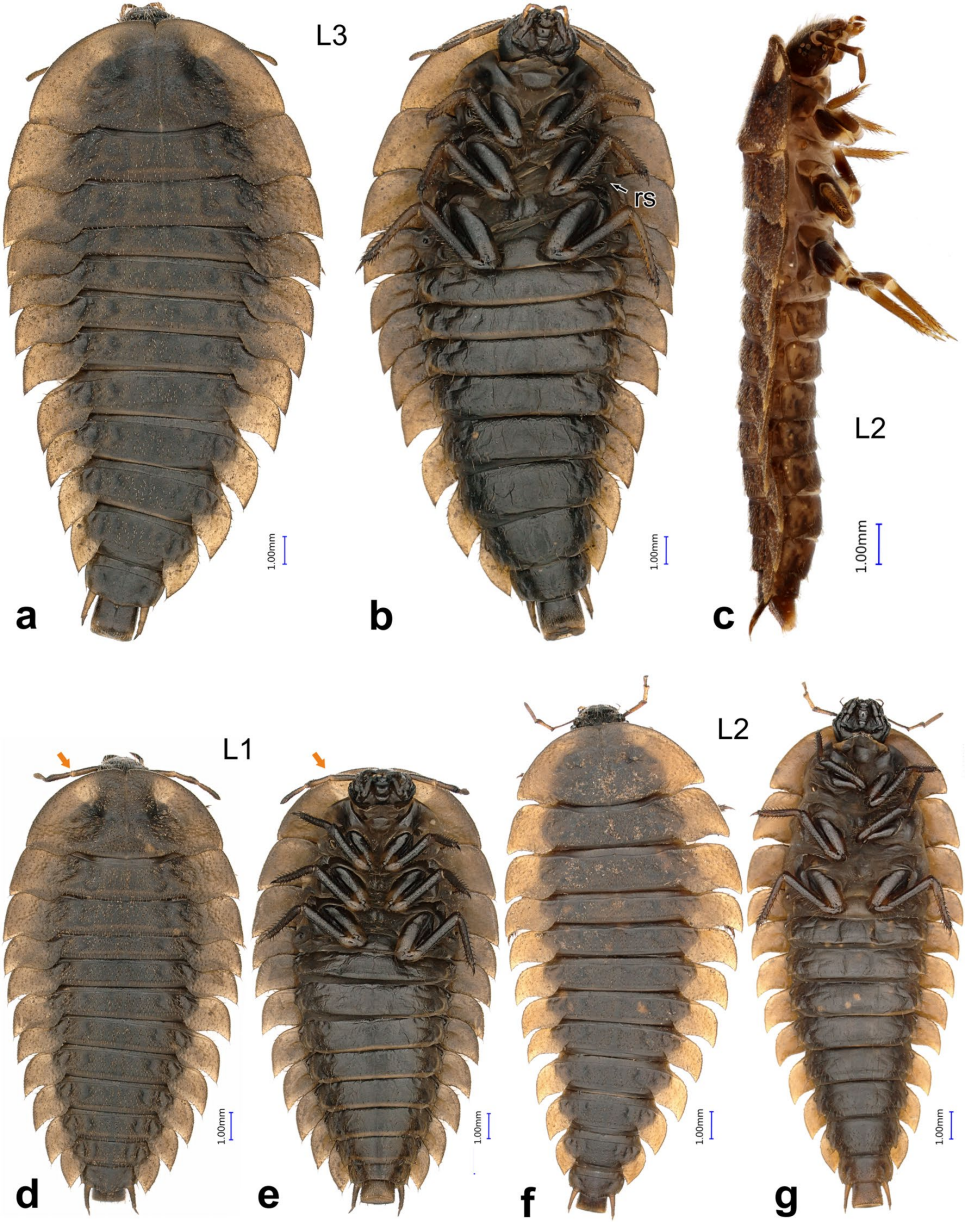
*Heterotemna tenuicornis* (Brullé, 1836), habitus of larvae: (**a**, **b**) third instar larva, dorsal and ventral view; (**c**) second instar larva, lateral view; (**d**, **e**) first instar larva, dorsal and ventral view; (**f**, **g**) second instar larva, dorsal and ventral view. Abbreviations: *rs* rudimentary spiraculum.

**Figure 4:**
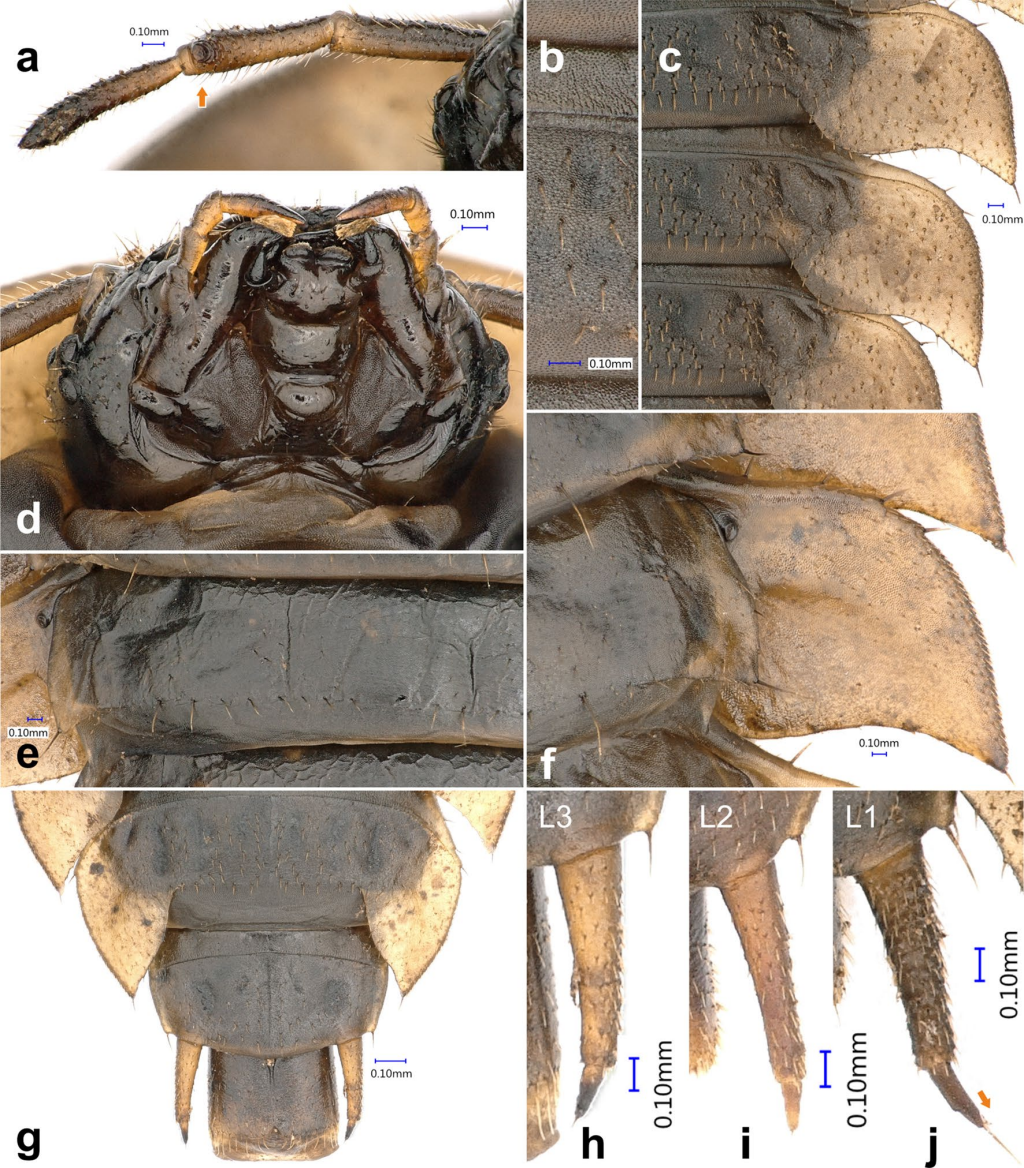
*Heterotemna tenuicornis* (Brullé, 1836), details of external morphology of larva: (**a**) antenna, dorsal view; (**b**) abdominal tergite, dorsal view; (**c**) abdominal tergites and paratergites, dorsal view; (**d**) head, ventral view; (**e**) abdominal ventrite, ventral view; (**f**) abdominal paratergite, ventral view; (**g**) abdominal tergites 8–10, dorsal view; (**h**–**j**) urogomphus, dorsal view, (**a**, **b**, **d**–**h**) third instar, (**i**) second instar, (**c**, **j**) first instar.

**Figure 5:**
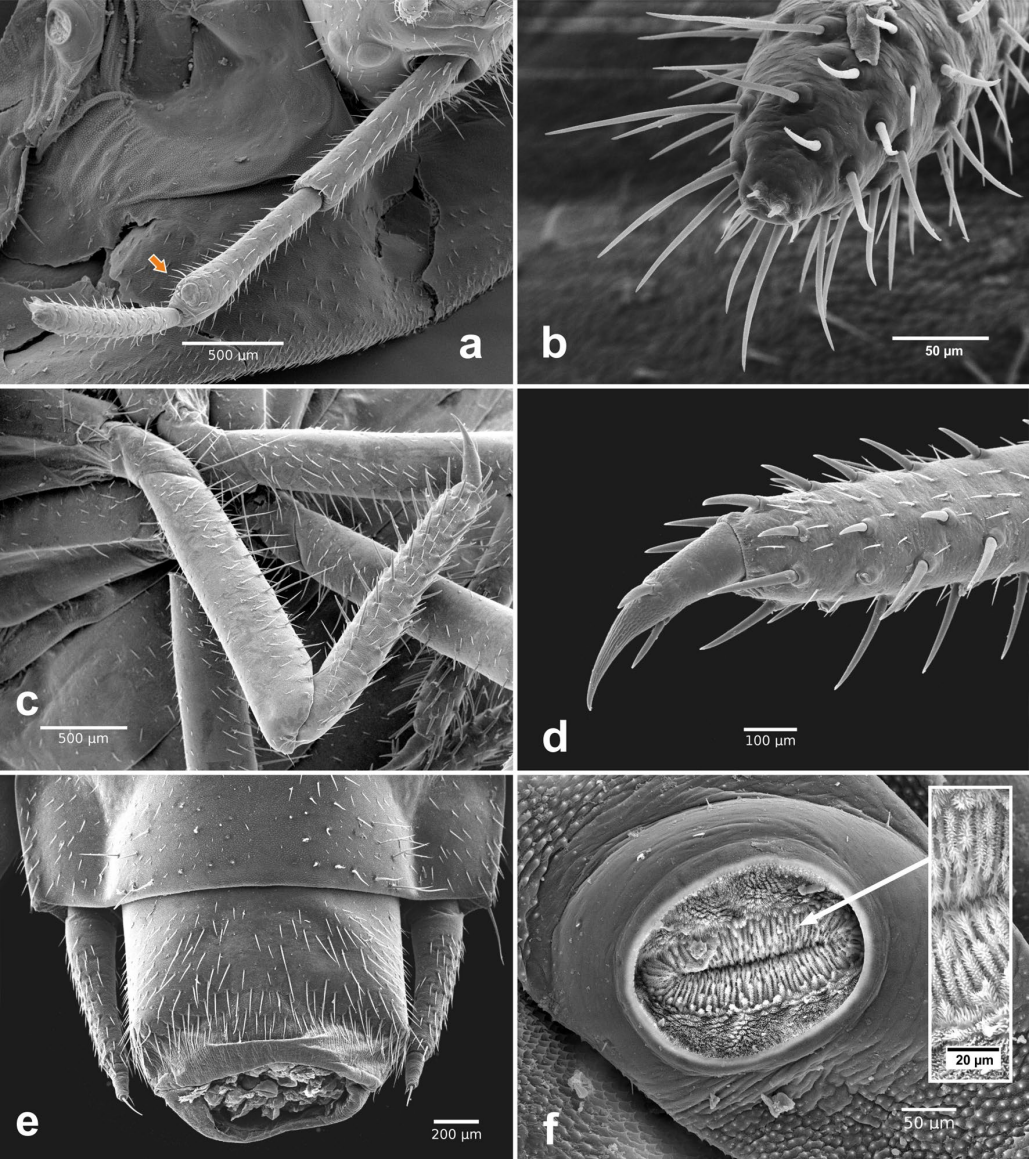
*Heterotemna tenuicornis* (Brullé, 1836), details of external morphology of larva, SEM: (**a**) antenna, dorsal view; (**b**) apex of antennomere III, dorso-apical view; (**c**) proleg, lateral view; (**d**) tibiotarsus and pretarsus, lateral view; (**e**) tergites IX–X and urogomphi, dorsal view; (**f**) mesosternal spiraculum in lateral view; inset—detail of multi-branched filtration hairs, (**a**, **c**–**f**) third instar, (**b**) first instar.

**Figure 6:**
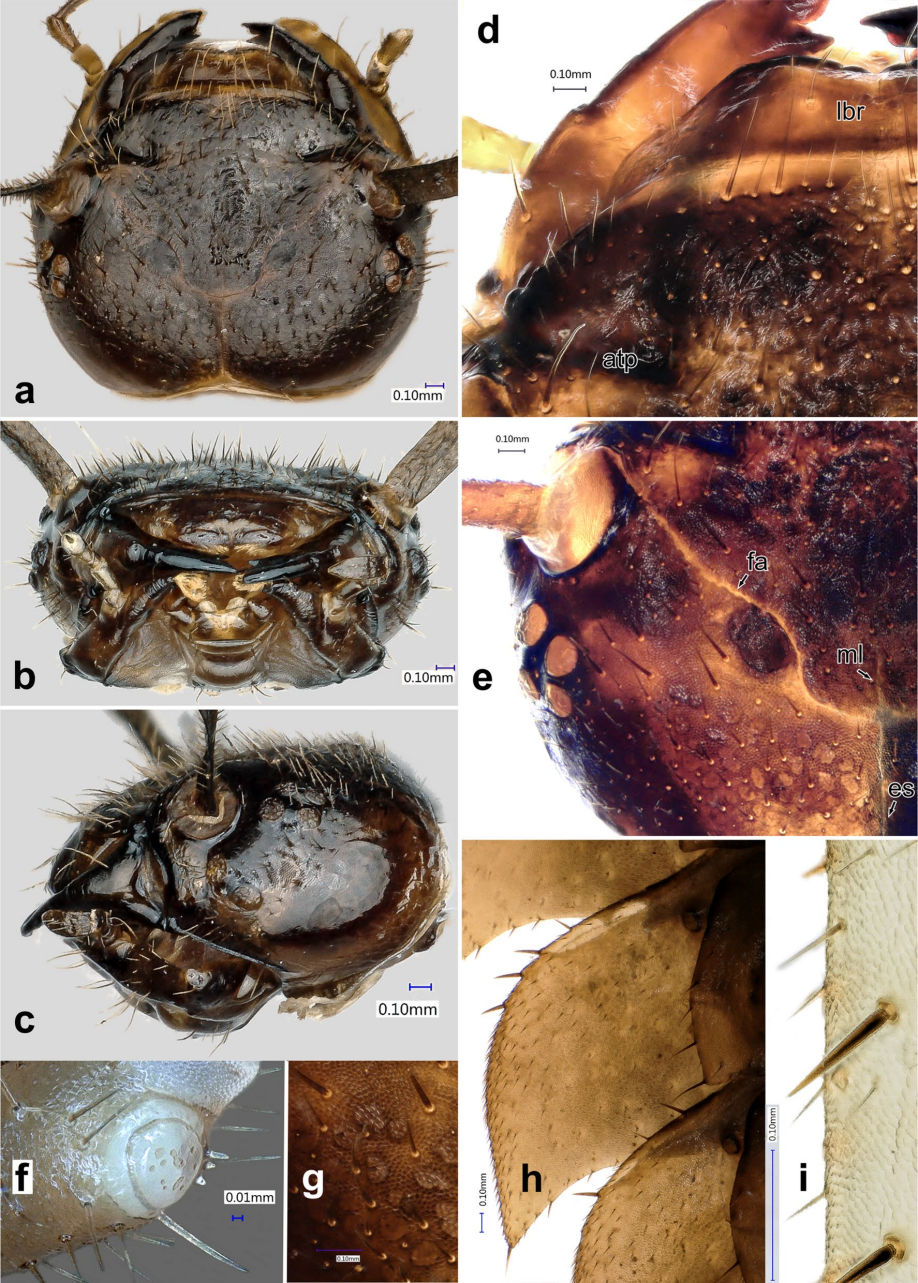
*Heterotemna tenuicornis* (Brullé, 1836), details of external morphology of larva, third instar: (**a**) head, dorsal view; (**b**) head, anterior view; (**c**) head, left lateral view; (**d**) detail of labrum and frons, dorsal view; (**e**) detail of epicranial plate with epicranial stem and frontal arms; (**f**) sensorium on antennomere II, dorso-lateral view; (**g**) epicranium, detail of surface, dorsal view; (**h**) abdominal paratergite, ventral view; (**i**) setation on tibiotarsus, lateral view. Abbreviations: *atp* anterior tentorial pits, *es* epicranial stem, *fa* frontal arms, *lbr* labrum, *ml* median line.

**Figure 7:**
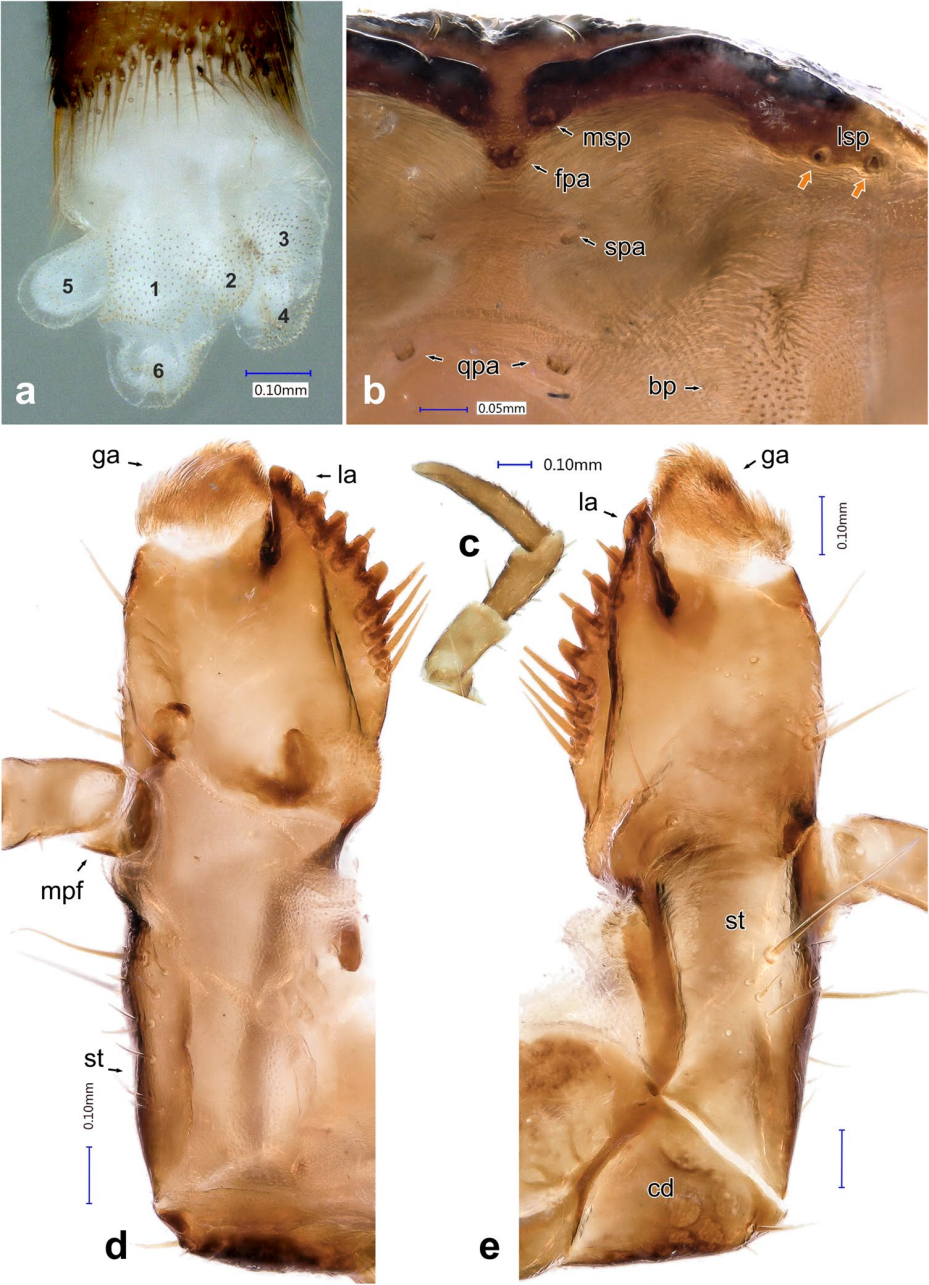
*Heterotemna tenuicornis* (Brullé, 1836), details of external morphology of larva, third instar: (**a**) segment X with six eversible lobes, ventrolateral view; (**b**) detail of epipharynx anteriorly, ventral view; (**c**) maxillary palpus, ventral view; (**d**) maxilla, dorsal view; (**e**) maxilla, ventral view. Abbreviations: *bp* bilobate pegs, *cd* cardo, *fpa* first porous area, *ga* galea, *la* lacinia, *lsp* lateral sensory pegs, *mpf* maxillary palpifer, *msp* median sensory peg, *qpa* quinque porous area, *spa* secondary porous area, *st* stipes.

**Figure 8:**
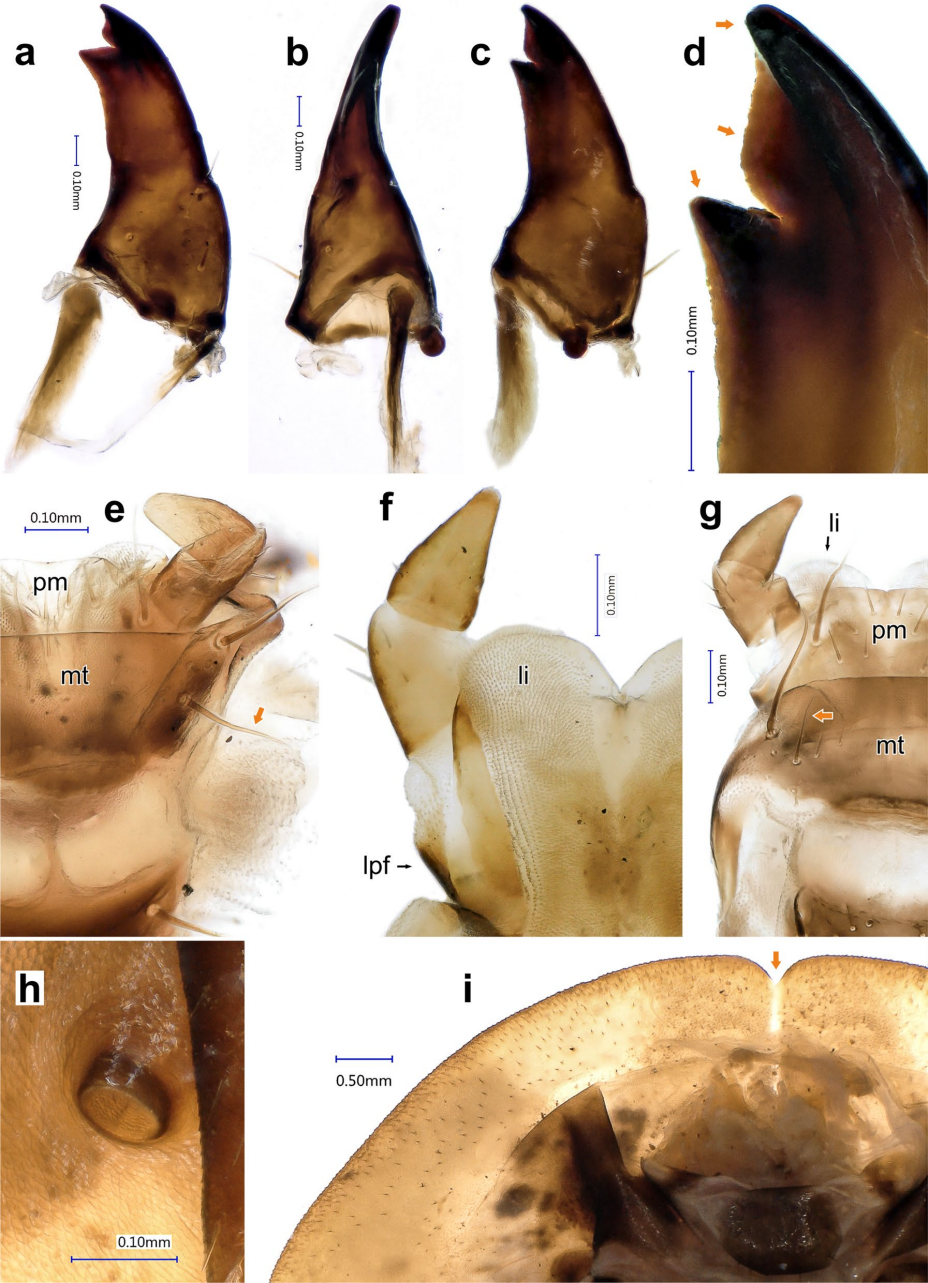
*Heterotemna tenuicornis* (Brullé, 1836), details of external morphology of larva: (**a**) right mandible, dorsal view; (**b**) left mandible, inner view; (**c**) left mandible, ventral view; (**d**) detail of apex, left mandible, ventral view; (**e**, **g**) labium, ventral view; (**f**) labium, dorsal view; (**h**) abdominal spiraculum, ventral view; (**i**) anterior part of protergum, ventral view, (**a**–**e**, **h**, **i**) third instar, (**f**–**g**) second instar. Abbreviations: *mt* mentum, *li* ligula, *lpf* labial palpifer, *pm* prementum.

Body teardrop-shaped (widest at front), distinctly dorsoventrally flattened, dark brown with lighter paratergites on thorax and abdominal segments I–VIII (Figs. 3a, d, f, 4c), with two lighter subcircular spots on protergum (Fig. 3a, d). Short median unsclerotized line extending beyond epicranial stem anteriorly (Fig. 6e). Epipharynx anterolaterally with two pairs of sensory pegs on its heavily sclerotized margin (Fig. 7b, lsp). Ventral epicranial ridges present, extending past the posterior edge of the hypostomal ridge (Fig. 4d). Antenna very slender and elongated (Figs. 4a, 5a). Antennomere II with large, round and flattened sensorium, bearing several sclerotized pores surrounded by narrow, sclerotized ring (Fig. 6f), externally with a wide unsclerotized area. More apically, antennomere II with three additional smaller sensilla (Figs. 4a, 5a, 6f). Anterior margin of protergum medially with narrow emargination (Fig. 8i). Rudimentary spiraculum present on metasternum (Fig. 3b). Paratergites of meso- and metathorax and abdominal segments I–VIII resembling the shape of pig ears, constricted posteriorly between tergite and paratergite, with apex pointed posteriorly (Figs. 3a, 4c, g). Ventrite II on abdomen entire, not subdivided into three sclerites (Fig. 3b).

#### Detailed description

*BodyInstar III.* Mean total body length: 16.65 mm ± 0.815 mm. Teardrop-shaped, fusiform to onisciform larvae, narrowing towards both ends, widest at metathorax (Fig. 3a–g), dorsoventrally distinctly flattened (Fig. 3c). Terga well-sclerotized and sparsely and regularly covered with short, recumbent setae (Fig. 4b, c). Dorsal surface dull, sparsely granulate, with regular isodiametric microsculpture (Fig. 4b). Paratergites of meso- and metathorax and abdominal segments I–VIII wide, resembling the shape of pig ears, constricted posteriorly between tergite and paratergite, with apex pointed posteriorly (Figs. 3a, 4c, g). Abdominal ventrites more glossy, without distinct microsculpture, posterior margin of ventrites with row of long, semierect setae (Fig. 4e). Dark brown pigmentation present on thoracic and abdominal sclerites, as well as on bases of paratergites. Remaining area of paratergites and lateral edges of protergum ochre (Fig. 4c, g). Laterally, tergites with darker brown spots and shallow depressions, arranged in two, slightly irregular, longitudinal rows; medially, dorsum with pale ecdysial line, reaching posteriorly to abdominal tergite IV. Additionally, pair of lighter subcircular translucent spots present on the frontal medial area of protergum, duller and not clearly delimited (Fig. 3a, b). *Instar II.* Pair of lighter subcircular translucent spots on protergum not clearly delimited (Fig. 3f–g). Mean total body length: 15.55 mm ± 1.16 mm. *Instar I.* Two lighter subcircular spots present on protergum more apparent, well-delimited (Fig. 3d–e). Mean total body length: 13.02 mm ± 0.77 mm.

*Head capsuleInstar III.* Prognathous. Head capsule covered dorsally and laterally by regular, densely arranged erected setation (Fig. 6a–c). Longest setae present on anterior and anterolateral part of frons (Fig. 6d–e). Cranium with isodiametric microsculpture, latero-posteriorly with paler spots (Fig. 6g). Epicranial stem (= coronal suture) present (Fig. 6a, es), V-shaped frontal arms (= frontal sutures) passing into U-shaped base in one third of their length (Fig. 6e, fa). Short median desclerotized line extending beyond epicranial stem (Fig. 6e, ml). Six stemmata on both sides of the head organized into two groups – four forming sub-rhomboid pattern placed dorsally behind antennal socket (Fig. 6e), and two ventro-laterally behind the antennal socket (Figs. 5a, 6c). Frontoclypeal suture absent, rudiments present only laterally as distinctly sclerotized, transverse, extension of anterior tentorial pits (Fig. 6d, atp). Clypeus trapezoidal; anterior margin distinctly sclerotized, widely emarginate and only narrowly desclerotized medially (Fig. 6b, d). Epipharynx, with the exception of medial part, covered by densely arranged microtrichia oriented posteriorly and medially (Fig. 7b). Anterolaterally, the heavily sclerotized epipharyngeal margin bearing two pairs of lateral sensory pegs (Fig. 7b, lsp). Anterior part emarginate, at its lateral angles with 1 pair of median sensory pegs (Fig. 7b, msp). Anteriorly, on first porous area, are two pairs of large sensilla (Fig. 7b, fpa). More posteriorly, secondary porous area consisting of two pairs of small sensilla (Fig. 7b, spa). More posteriorly, near base, with broad, weakly arched parabolic row of 18 pores (or cibarial plates), bounded posteriorly on each side by group of 5 pores (quinqueporous area) (Fig. 7b, qpa). Two pairs of bilobate pegs located more laterally (Fig. 7b, bp). Hypopharynx membranous, with transverse hypopharyngeal bracon. Tentorium consisting of pair of sclerotized anterior arms, laterally narrowly extended by fine hyaline lobes in their basal 2/3, before the dorsal arms connecting to them; hyaline dorsal arms connected to frons near the beginning of the U-shaped base of the frontal arms; and sclerotized posterior arms connected to broad posterior tentorial bridge. Ventral epicranial ridges present, extending past the posterior edge of hypostomal ridge (Fig. 4d). Hypostomal rods absent. Gular region very short, with gular sutures converging anteriorly. Head size: HW 2.842 mm ± 0.077 mm. *Instar II.* Head size: HW 2.327 mm ± 0.075 mm. *Instar I.* Head size: HW 1.927 mm ± 0.045 mm.

*Mandibles* (Fig. 8a–d) *Instar III.* Symmetrical (Fig. 6b), simple without mola or prostheca. Apical tooth longer than sub-apical tooth, apex regularly bent dorsally in inner lateral view (Fig. 8b). Two stout setae present dorsally and dorso-laterally on mandibular base; additional short, stout seta present dorsally in the mid-length of the mandible. Inner margin of both apical and subapical teeth finely serrate (Fig. 8d). *Instar II* and *Instar I* same as Instar III.

*Antenna* (Figs. 4a, 5a, 6f) *Instar III.* Trimerous and fully sclerotized, inserted in membranous socket on lateral distal margin of genal region. Sensorium of AII placed on outer lateral area of its distal end, consisting of round, flattened bulb covered with several large pores, and belted by sclerotized ring and three small sensilla arranged in triangle adjacent posteriorly to the sensorium (Fig. 6f). All three antennomeres densely covered by stout setae across the surface (Fig. 5a). Apex of AIII with three articulated sensilla and one elongate, slender seta (as on Fig. 5b). Antennomere proportions: first two segments slightly shorter than third segment (Fig. 4a); AI 1.213 mm ± 0.138 mm, AII 0.912 mm ± 0.009 mm, and AIII 0.989 mm ± 0.164 mm. *Instar II.* Antennomere proportions: AI (1.214 mm ± 0.215 mm) slightly longer than AII (1.031 mm ± 0.15 mm) and AIII (1.093 mm ± 0.117 mm). *Instar I.* Antennomere proportions: AI (0.713 mm ± 0.083 mm) and AII (0.07 mm ± 0.041 mm) of similar length and AIII on average slightly longer (0.824 mm ± 0.06 mm).

*Maxilla* (Fig. 7c–e) *Instar III.* Attached closely laterally to labium. Cardo broad (Fig. 7e, cd), separated in ventral view into two parts by narrow, heavily sclerotized, longitudinal apodeme (joined anteriorly to inner margin of stipes). Base of cardo with one large seta placed on outer ventral margin and two smaller setae, one placed dorsally and another ventrally. Stipes elongate, subrectangular; ventral side regularly sclerotized, with more distinctly sclerotized apodeme on inner margin, joining cardo with inner base of lacinia (Fig. 7e, st). Ventral surface with group of several short setae and one large seta in the middle of the length. Outer lateral margin with another small seta and additional single large seta, placed more anteriorly. Dorsal surface only narrowly sclerotized along outer margin, with irregular row of short setae and pores (Fig. 7d, st). Most of dorsal surface weakly sclerotized. Lacinia and galea basally fused, separated only apically (Fig. 7d–e). Sclerotized basal fused part ca. 1.4 as long as wide. Distinct cuticular spines present on inner lateral and dorsal base of lacinia; base in dorsal view with two distinctly sclerotized, slightly dorsally elevated regions (Fig. 7d). Fused basal part on outer margin in ventral view with two large, laterally pointing setae, and several small setae and pores. Another two large setae present at base. Free apical part of lacinia distinctly sclerotized, with elongate longitudinal apodeme in ventral view. Inner margin with heavily sclerotized lobes, with 9–11 large, stout teeth. Lacinia elongate apically, apex shorter than galea. Galea with a compact, relatively small brush of setae (Fig. 7d, e, ga). Maxillary palpus trimerous, short basal palpifer present (Fig. 7c, mpf). Third segment cylindrical, apically with group of sensilla. Proportions of maxillary palpomeres: MPI 0.248 mm ± 0.009 mm, MPII 0.277 mm ± 0.057 mm, MPIII 0.468 mm ± 0.026 mm. *Instar II:* Basal fused part of lacinia and galea shorter and wider, sclerotized part only slightly longer than wide. Galea with large brush of setae. Proportions of maxillary palpomeres: MPI 0.189 mm ± 0.032 mm, MPII 0.245 mm ± 0.024 mm, MPIII 0.396 mm ± 0.087 mm. *Instar I:* Basal fused part of lacinia and galea short and wide, sclerotized part as long as wide. Galea with large brush of setae. Proportions of maxillary palpomeres: MPI 0.158 mm ± 0.041 mm, MPII 0.199 mm ± 0.029 mm, MPIII 0.384 mm ± 0.067 mm.

*Labium* (Fig. 8e–g) *Instar III.* Submentum present ventrally at base of maxillo-labial complex aslarge, broadly trapezoidal sclerite, posterolateral corners extending to base of cardo, and to transversely oriented posterior tentorial pits (Fig. 4d). Anterior part weakly sclerotized, distinctly separated from mentum. Surface postero-laterally with two pairs of large setae, surface irregularly covered by several additional short setae. Mentum wide, sclerotization extended also laterally, with two pairs of very large setae positioned laterally, surrounded by additional several short setae and pores (Fig. 8e, mt). Prementum transverse, narrowing anteriad, irregularly sclerotized. Laterally with one pair of large setae, medially with several small setae (Fig. 8e, pm). Ligula bilobed, heart-shaped, sclerotized laterally and basally, each lobe along the sagittal plane covered dorsally by numerous longitudinal lines of fine short setation and dense bulbous projections apically and centrally between the two lobes (as on Fig. 8f, li). Labial palpus bimerous; basally with longitudinal, laterally sclerotized palpifer (as on Fig. 8f, lpf). Basal palpomere club-shaped, laterally with four and ventrally with two short setae, distal palpomere conical, with several very short setae and several sensilla laterally and apically. *Instar II.* Mentum ventrally with one pair of very long and one pair of slightly shorter setae laterally (Fig. 8g). *Instar I.* Setae on mentum as in instar II.

*Thorax* (Fig. 3a, b) *Instar III.* Three-segmented. Protergum sub-semicircular (Figs. 3a, 8i). N1W 8.633 mm ± 0.283 mm, protergum widest at base with narrow emargination at medial part of anterior margin (Fig. 8i). Pair of subcircular lighter patches present anteriorly, only vaguely delimited (Fig. 3a). Mesotergum wider than protergum, metatergum being the widest part of the body (Fig. 3a). Paratergites of meso- and metatergum distinctly longer than paratergites on abdomen (Fig. 3a, b), both segments also distinctly more robust than abdominal segments (Fig. 3a). Venter of prothorax composed of prosternum, subdivided into three semi-sclerotized areas; lateral ones small; medial area largest. Ventrolateral areas of pro-, meso-, and metathorax composed of episternum and epimeron with short pleural suture in between them and well-sclerotized pre- and postcoxale. Meso- and metasternum subdivided by transverse fold into membranous basisternum and sternellum; basisterna on both segments medially with well sclerotized areas, sagittally divided into two plates (Fig. 3b). Laterotergites poorly sclerotized, with mesothoracic spiraculum opened, its inner part densely covered with multi-branched filtration hairs (Fig. 5f). Rudimentary spiraculum also present on laterotergites of metasternum (Fig. 3b, rs). *Instar II:* N1W 6.909 mm ± 0.340 mm. *Instar I*: Pair of subcircular lighter patches well-delimited (Fig. 3d). N1W 5.713 mm ± 0.237 mm.

*Legs* (Fig. 5c) Pentamerous, including pretarsus, relatively elongated (femur ca. 4 times as long as wide); regularly covered with two types of setae—short and thin and long and stout (Figs. 5d, 6i). Surface with regular, isodiametric microsculpture (Figs. 5d, 6i). Femur ventrally covered with additional row of longer setae (Fig. 5c). Tibiotarsus covered regularly with longer, stout setae (Fig. 5d). Tarsal claw with pair of opposed setae (Fig. 5d).

*Abdomen* (Figs. 3a, b, 4b, c, e–g, 5e, 6h): *Instar III* Ten-segmented. Tergites regularly narrowing posteriad, length of segments slightly increasing towards abdominal apex (Fig. 3a). Paratergites I–IX covered with minute setae, antero-lateral margin with four larger setae, posterior apex with single, large seta (Figs. 4f, 6h). Anterior part ventrally with distinctly sclerotized spiraculum, opened postero-laterally (Fig. 8h). Ventrite I reduced, present only medially, lateral portions largely unsclerotized. Ventrite II entire, similar to ventrites III–IX, not broken into three sclerites (Fig. 3b). Tergite IX subrectangular, with reduced paratergites, with two posteriorly oriented apical setae and with well-developed pair of two segmented urogomphi (Figs. 4g, 5e). Basal segment of urogomphi densely covered by recumbent setae; only scarce setae present ventrally on distal segment (Figs. 4h, 5e). Segment X subcylindrical, holding pygopod with six eversible lobes, covered by fine spines (Figs. 5e, 7a). Distal edge covered with row of posteriorly oriented setae (Fig. 4g). First (basal) urogomphal segment (URI 1.006 mm ± 0.065 mm) widest at its base, narrowing posteriorly, shorter than segment X (Fig. 4h). Second segment conical, narrowing towards its end (URII 0.234 mm ± 0.019 mm) with short stout seta (US 0.143 mm ± 0.013 mm) (Figs. 4h, 5e). *Instar II*: First segment of urogomphi longer than segment X (Fig. 4i), URI 0.803 mm ± 0.051 mm, URII 0.188 mm ± 0.033 mm and US 0.152 mm ± 0.021 mm. *Instar I:* First segment of urogomphi longer than segment X (Fig. 4j), URI 0.621 mm ± 0.064 mm, URII 0.208 mm ± 0.037 mm and US 0.29 mm ± 0.044 mm.

### Identification key to instars of *Heterotemna tenuicornis*

1. Pair of lighter subcircular translucent spots on frontal medial area of protergum more distinct, well-delimited (Fig. 3d, e). Head width 1.83–2.01 mm. Protergum width 5.24–6.19 mm. … **First instar**
2. Pair of lighter subcircular translucent spots present on frontal medial area of protergum, duller and not clearly delimited (Fig. 3a, b, c, e). Head width 2.19–2.91 mm. Protergum width 6.38–9.11 mm^2^.
3. Head width 2.19–2.43 mm. Protergum width 6.38–7.47 mm. First segment of urogomphi longer than segment X (Fig. 4i). … **Second instar**
4. Head width 2.72–2.91 mm. Protergum width 8.39–9.11 mm. First segment of urogomphi shorter than segment X (Fig. 4h). … **Third instar**

## Discussion

Our study reports the first DNA sequences of the genus *Heterotemna*, allowing molecular identification of the genus. This can be useful, especially if dealing with incomplete specimens or stages that do not allow morphological identification, such as eggs and pupae. Based on the DNA sequences, we were able to infer the phylogenetic placement of the genus within the subfamily Silphinae. The phylogenetic tree presented in this study is mostly in agreement with the current molecular phylogeny of the subfamily^26,29,30^. The aim of our study was not to reconstruct the phylogeny of the entire Silphinae, but provide a tentative placement of the genus *Heterotemna*. For this purpose, we used representatives of several recognized genera—*Ablattaria*, *Necrodes*, *Oiceoptoma*, *Phosphuga*, *Silpha*, and *Thanatophilus.* Our results show that the monophyly of the genera *Necrodes*, *Oiceoptoma*, and *Thanatophilus* is well supported and in accordance with previous works. In our study we also observed a well-supported clade that consists of the genera *Ablattaria*, *Heterotemna*, *Phosphuga*, and *Silpha*. As the general topology of the presented phylogenetic tree is in agreement with previous studies, we assume that it can be considered a reliable estimate of the evolutionary relationships of the genus *Heterotemna* to other silphine genera. The monophyly of the clade containing *Ablattaria*, *Aclypea* Reiter, 1885, *Dendroxena* Motschulsky, 1858, *Silpha*, and *Phosphuga* is well supported^26,29,30^. However, relationships and taxonomic positions of genera inside the internal group of the subfamily Silphinae^26^ are unresolved and there is an ongoing discussion regarding the genera *Phosphuga* and *Ablattaria* which are either considered subgenera of the genus *Silpha* or separate monophyletic genera^24,27^. Furthermore, evidence for paraphyly of the genus *Silpha* with respect to the genus *Aclypea* was indicated by the previous phylogenetic study^29^. Our results add up more evidence toward suggested paraphyly also with the respect to the genus *Heterotemna*. The most pragmatic taxonomic solution would to treat *Heterotemna* as a junior synonym of *Silpha*. To fully resolve the issue is out of scope of our study as more robust phylogeny at the genus level is needed. Such a step will require the inclusion of additional *Silpha* species, e.g., possibly closely related *S. puncticollis*, and other species of the internal group.

Based on the phylogenetic tree presented in this study, *Silpha tristis* was placed as a sister species to *H. tenuicornis. Silpha tristis* is a widespread species in the Western Palaearctic region, also recorded in North Africa (Morocco)^31^ but not present on the Canary Islands. The only *Silpha* species occurring in Tenerife is the introduced *Silpha puncticollis*, a western Mediterranean species^28^ possibly closely related to *S. tristis*. The effect of the presence of *S. puncticollis* on endemic species of *Heterotemna* has not been studied. However, as there is very limited information on the ecology and biology of both species, we believe, that it is crucial to be able to recognize all life stages of the endemic species from the newly introduced one. Adults of these two species are easily distinguished from each other by dense, fine and uniformly punctured pronotum with thick and reflexed borders, and the absence of protuberances or carina on its disk, and elytra with elevated costae in *S. puncticollis*^28,32^, and disk with a pair of median longitudinal carinae and elytra with much finer costae in *Heterotemna*^25^ (Fig. 2a). However, the morphology of larvae of genus *Heterotemna* has not been previously described and we hereby provide the first clear detailed description.

The larvae fit the general body plan and features typical for the larva of Silphinae, summarized as^33^: relatively large (usually over 12 mm in L3); body slightly to strongly flattened, body surface heavily pigmented and sclerotized; head with 6 pairs of stemmata; mandible without a molar lobe or prostheca; maxilla with broad, apically cleft basal fused part bearing galea with dense setal brush on outer lobe; urogomphi articulated, usually 2-segmented.

The larva of *Heterotemna* differs from other known larvae of *Silpha* as follows: (1) protergum with two lighter subcircular spots anterolaterally on disc; anterior margin medially with narrow emargination (in *Silpha*, protergum differently coloured: unicolour or with paler postero-lateral part; anterior margin simple, regularly rounded or very widely emarginated—see^34^ for details). However, the larva of *S. puncticollis* is unknown.

In our study we did not observe an overlap in head width and protergal width between all three instars. Variation in larval head and protergal width was observed across Silphinae^35,36^ for example in *Thanatophilus*^38^, therefore, it seems that by using these two measurements, instars of *H. tenuicornis* can be reliably distinguished. In addition, the pair of lighter spots on the protergum seemed to be also one of the characteristics to distinguish between instars (being most contrasted and sharply delimited in first instar larvae than in second and third instar). However, larvae of all three instars that were available for this study varied in date of collecting and some of the specimens were collected as long as 13 years ago, therefore, we are cautious as the colours may have changed during storage, as can be seen in other species of Silphinae (M. Novak, unpubl. data). Therefore, we focused mainly on morphological structures and use the coloration as secondary trait of instar determination. Larvae of *H. tenuicornis* seem to share some morphometric characters with other species of the subfamily Silphinae. There is a general pattern of abrupt increase of the ratio between the length of the first and the second segment of the urogomphi as well as between the second segment of the urogomphi and the terminal seta when comparing first and second instars. A similar pattern was also observed in two species of the genus *Thanatophilus*^37,38^ and our preliminary observations also confirm this for larvae of *Diamesus osculans* (Vigors, 1825).

In order to obtain more information regarding the biology and ecology of the genus *Heterotemna* we created a basic identification key, that could encourage data collecting. Identification based on morphology of focal species is a crucial tool when it comes to collecting information in the field as well as studying the ecology and adaptations of the species. It is still an irreplaceable discipline that cannot be neglected or replaced, even by fast-evolving molecular methods.

## Materials and methods

### Specimen sampling

Specimens were collected in 2007, 2011 and 2017 (SM3) with pitfall traps, and killed and stored in 75% or 96% ethanol. In total, we obtained 48 individuals for further morphological examination. Only the specimens stored in 96% ethanol were used for phylogenetic analysis. As the larvae were collected in the wild and were not raised to adults, the species identification could not be confirmed morphologically. Therefore, we obtained a single adult specimen of *H. tenuicornis* to confirm the molecular identification of the larvae as the same species. Genetic distance was calculated using the COI and 16S genes among all taxa used for the phylogenetic analysis, and the adult specimen of *H. tenuicornis* was used to confirm the conspecificity of the larval *H. tenuicornis* using DNADIST version 3.5c implemented in BioEdit v7.0.5.3^39^.

### Morphological analysis

The morphological terminology used in this paper follows Lawrence and Ślipiński^40^ and Novák et al.^38^. The terminology of the epipharynx follows Dorsey^41^ and Anderson^42^. Morphological characters were measured, documented, and examined using a Keyence VHX-6000 digital microscope. To observe detailed structures located within the head capsule, the head was detached and submerged in hot (90 °C) 10% potassium hydroxide (KOH) for 3 min. The head capsule was subsequently dissected and detailed structures of the epipharynx and the head appendages were observed.

To observe very fine structures such as setae and pores, we used a scanning electron microscope. Preparation of samples follows the methodology of Novák et al.^38^. Selected specimens were dehydrated using a graded series of ethanol (75%, 80%, 90%, 95%, 100%) and left in each concentration for approximately 30 min. before transferring to acetone overnight. Dehydrated samples were dried using the critical point drying method. Dry samples were then attached to an aluminium disk target using copper foil tape and coated with gold in Bal-Tec Sputter Coater SCD 050. Samples were observed and documented with a JSM-6380LV (JEOL) scanning electron microscope.

The following morphological characters were measured: The length of the first antennomere (AI), the length of the second antennomere (AII), protergal width (N1W), head width (at the widest point) (HW), length of the first urogomphal segment (UI), length of the second urogomphal segment (UII), length of urogomphal seta (when present) (US II), length of all three palpomeres (MPI, MPII, MPIII). The morphological characters are described based on the third instar (L3) larvae followed by observed differences in second (L2) and first instar larvae (L1). The mean and standard deviation (± SD) of characteristics measured is indicated in the text.

The changes in the sizes of the measured morphological characters and their ratios throughout the developmental stages were tested using a linear model with a normal distribution of errors. The significance level was set at 5%. The analysis was carried out in R program (R Core Team 2020). Graphical outputs were created using ggplot2 and sjPlot packages^43,44^.

### Molecular analysis

Genomic DNA was extracted using commercial Tissue & Blood Kit (Geneaid, New Taipei City, Taiwan) following the protocol provided. Two mitochondrial genes were partially amplified—Cytochrome oxidase I (COI) using the primer pair “Jerry” 5′-CAACATTTATTTTGATTTTTTGG-3′ and “Pat” 5′-TCCAATGCACTAATCTGCCATATTA-3′^45^ and 16S (rDNA) using following primer pairs “LR-J-12887” 5′-CTC CGG TTT GAA CTC AGA TCA-3′ and “LR-N-13398” 5′-CGC CTG TTT ATC AAA AAC AT-3′^45^ and “16SL” 5′-ATT CTA AAT YYA WNG CAC TAW TCT GCC AAA-3′^46^ and “16SAH” 5′-YGC CTG TTT AWY AAA AAC ATG-3′^47^. The concentrations of reagents for premix for PCR was based on PPP Master Mix (Top-Bio), the PCR reactions were carried out at 25 µl based on provided protocol (12.5 µl of 1 × PPP Master Mix, 9.5 µl PCR H_2_O, 0.4 μM of forward and 0.4 μM reverse primer) under the following conditions: COI: initial denaturation 94 °C for 3 min, followed by 35 cycles of 94 °C for 30 s, 50 °C for 30 s and 72 °C for 2 min and final extension at 72 °C for 10 min; 16S (LR primers): initial denaturation at 94 °C for 3 min, followed by 35 cycles of 94 °C for 30 s, 51 °C for 30 s and 72 °C for 45 s and final extension 72 °C for 10 min; 16S SAH primers: initial denaturation at 94 °C for 3 min, followed by 35 cycles of 94 °C for 30 s, 56 °C for 30 s and 72 °C for 45 s and final extension 72 °C for 10 min. PCR products were visualized by electrophoresis on a 1.5% agarose gel. PCR products were purified using ExoSAP-IT (Applied Biosystems) (following the protocol provided), sequencing was carried out in BIOCEV (Vestec, Czech Republic). Sequencing was performed in both directions using the same primes as for PCR. Newly generated sequences used in this study were obtained from two larvae and one adult specimen of *H. tenuicornis* and adults of *Thanatophilus mutilatus* (Laporte de Castelnau, 1840), *Ablattaria laevigata* (Fabricius, 1775), *Phosphuga atrata* (Linnaeus, 1758), *Silpha carinata* Herbst, 1783, *Silpha obscura* Linnaeus, 1758, *Silpha olivieri* Gebler, 1832, and *Silpha tristis* Illiger, 1798 stored in 96% EtOH. The GenBank accession numbers of newly generated sequences are available in SM2.

### Phylogenetic analyses

The electropherograms obtained were proofread and corrected for miss-called bases in Chromas 2.6.6. (Technelysium Pty Ltd, South Brisbane, Australia). Additional sequences used for the phylogenetic analyses were obtained from GenBank (National Centre for Biotechnology Information, https://www.ncbi.nlm.nih.gov/genbank/) (SM2). Multiple sequence alignments were generated with MAFFT version 7^48^ using The Guidance2 Server^49^. Aligned sequences were further manually edited in BioEdit 7.0.5.3^39^. Concatenated sequences consisting of 16S and COI were analysed under the criterion of maximum parsimony (MP) using PAUP 4.0a^50^. The MP analysis was conducted with heuristic search and 10,000 bootstrap replicates. The GTR + I + G evolutionary model was selected in jModelTest 2^51,52^ for both genes (16S and COI) using the Akaike Information Criterion (AIC)^53^. The tree topology was estimated using Bayesian phylogenetic inference (BI) based on selected evolutionary model (GRT + I + G; invgamma) for both partitions using MrBayes 3.1.2. software^54,55^. The search was conducted for two simultaneous runs with four independent chains for 10,000,000 generations, sampled every 1000 generations, the average standard deviation of split frequencies reached 0.002093. The first 25% generated trees from both runs were discarded as burnin. Maximum likelihood (ML) analysis was conducted using IQ-TREE web server^56^ based on GTR + I + G model and 10,000 bootstrap replicates. Additionally, COI and 16S sequences obtained from an adult specimen were compared to relevant sequences from larval specimens in MEGA 10.1.07^57^, sequences were analysed among and within groups (group *Silpha*: *S. tristis*, *S. obscura*, *S. perforata*, *S. carinata*, *S. olivieri* and group *Heterotemna: H. tenuicornis* (Larva) 1, *H. tenuicornis* (Larva) 2, *H. tenuicornis* (Adult)) using K2P model. The outgroups, two species of the family Staphylinidae, were selected based on previous studies: *Scaphidium quadrimaculatum* (subfamily Scaphidiinae)^30^ and *Aleochara curtula* (subfamily Aleocharinae)^29^.

## Supplementary Information

Supplementary Information.

## Data Availability

We provide following data used in our study—Measurements and ratios of all three larval stages of *H. tenuicornis* (in millimetres) (SM4), GenBank accession numbers of all sequences used in our study (SM2), including newly generated sequences that have been submitted in GenBank.
