# Supplementary material for: Description of larval morphology and phylogenetic relationships of Heterotemna tenuicornis (Silphidae)
Source: Sci Rep. 2021 Aug 20;11:16973. doi: 10.1038/s41598-021-94744-x (PMC8379240; doi:10.1038/s41598-021-94744-x)
Supplement: Supplementary file 1 — Supplementary Information. [file 41598_2021_94744_MOESM1_ESM.pdf]

SM1: Calculated distances between concatenated COI and 16S sequences of all studied specimens.

	Silpha carinata	*Silpha perforata*	*Silpha obscura*	*Silpha olivieri*	*Heterotemna tenuicornis* 116 Adult	*Heterotemna tenuicornis* 113 Larva	*Heterotemna tenuicornis* 112 Larva	*Silpha tristis*	*Oiceoptoma subrufum*	*Oiceoptoma nigropunctatum*	*Necrodes nigricornis*	*Phosphuga atrata*	*Ablattaria laevigata*	*Necrodes littoralis*	*Thanatophilus rugosus*	*Thanatophilus sinuatus*	*Thanatophilus capensis*	*Aleochara curtula*	*Scaphidium quadrimaculatum*
Silpha carinata	*0.0000*	*0.0664*	*0.0794*	*0.0752*	*0.0870*	*0.0848*	*0.0847*	*0.0941*	*0.1160*	*0.1066*	*0.1237*	*0.1049*	*0.0827*	*0.1197*	*0.1276*	*0.1094*	*0.1264*	*0.1921*	*0.2031*
Silpha perforata	*0.0664*	*0.0000*	*0.0838*	*0.0907*	*0.0904*	*0.0904*	*0.0903*	*0.0964*	*0.1149*	*0.1101*	*0.1273*	*0.1093*	*0.0871*	*0.1276*	*0.1419*	*0.1231*	*0.1321*	*0.1922*	*0.2100*
Silpha obscura	*0.0794*	*0.0838*	*0.0000*	*0.0445*	*0.0753*	*0.0720*	*0.0709*	*0.0824*	*0.1069*	*0.1080*	*0.1166*	*0.0877*	*0.0859*	*0.1308*	*0.1293*	*0.1082*	*0.1195*	*0.1713*	*0.1865*
Silpha olivieri	*0.0752*	*0.0907*	*0.0445*	*0.0000*	*0.0722*	*0.0700*	*0.0689*	*0.0814*	*0.0977*	*0.1077*	*0.1119*	*0.0846*	*0.0770*	*0.1106*	*0.1175*	*0.1081*	*0.1255*	*0.1669*	*0.1831*
Heterotemna tenuicornis 116 Adult	*0.0870*	*0.0904*	*0.0753*	*0.0722*	*0.0000*	*0.0078*	*0.0068*	*0.0700*	*0.0870*	*0.0947*	*0.1280*	*0.0861*	*0.0811*	*0.1276*	*0.1334*	*0.1146*	*0.1319*	*0.1706*	*0.1983*
Heterotemna tenuicornis 113 Larva	*0.0848*	*0.0904*	*0.0720*	*0.0700*	*0.0078*	*0.0000*	*0.0029*	*0.0657*	*0.0847*	*0.0924*	*0.1244*	*0.0829*	*0.0778*	*0.1253*	*0.1335*	*0.1158*	*0.1261*	*0.1710*	*0.1988*
Heterotemna tenuicornis 112 Larva	*0.0847*	*0.0903*	*0.0709*	*0.0689*	*0.0068*	*0.0029*	*0.0000*	*0.0656*	*0.0846*	*0.0923*	*0.1255*	*0.0840*	*0.0777*	*0.1252*	*0.1310*	*0.1146*	*0.1283*	*0.1734*	*0.2000*
Silpha tristis	*0.0941*	*0.0964*	*0.0824*	*0.0814*	*0.0700*	*0.0657*	*0.0656*	*0.0000*	*0.0806*	*0.0917*	*0.1239*	*0.0924*	*0.0846*	*0.1201*	*0.1227*	*0.1182*	*0.1200*	*0.1698*	*0.1949*
Oiceoptoma subrufum	*0.1160*	*0.1149*	*0.1069*	*0.0977*	*0.0870*	*0.0847*	*0.0846*	*0.0806*	*0.0000*	*0.0392*	*0.1114*	*0.1015*	*0.0880*	*0.1125*	*0.1179*	*0.1002*	*0.0997*	*0.1534*	*0.1836*
Oiceoptoma nigropunctatum	*0.1066*	*0.1101*	*0.1080*	*0.1077*	*0.0947*	*0.0924*	*0.0923*	*0.0917*	*0.0392*	*0.0000*	*0.0975*	*0.1149*	*0.0958*	*0.1107*	*0.1232*	*0.1070*	*0.1114*	*0.1653*	*0.1933*
Necrodes nigricornis	*0.1237*	*0.1273*	*0.1166*	*0.1119*	*0.1280*	*0.1244*	*0.1255*	*0.1239*	*0.1114*	*0.0975*	*0.0000*	*0.1342*	*0.1182*	*0.0976*	*0.1298*	*0.1110*	*0.1227*	*0.1708*	*0.1915*
Phosphuga atrata	*0.1049*	*0.1093*	*0.0877*	*0.0846*	*0.0861*	*0.0829*	*0.0840*	*0.0924*	*0.1015*	*0.1149*	*0.1342*	*0.0000*	*0.0872*	*0.1255*	*0.1233*	*0.1256*	*0.1299*	*0.1739*	*0.2019*
Ablattaria laevigata	*0.0827*	*0.0871*	*0.0859*	*0.0770*	*0.0811*	*0.0778*	*0.0777*	*0.0846*	*0.0880*	*0.0958*	*0.1182*	*0.0872*	*0.0000*	*0.1158*	*0.1189*	*0.1134*	*0.1188*	*0.1722*	*0.2012*
Necrodes littoralis	*0.1197*	*0.1276*	*0.1308*	*0.1106*	*0.1276*	*0.1253*	*0.1252*	*0.1201*	*0.1125*	*0.1107*	*0.0976*	*0.1255*	*0.1158*	*0.0000*	*0.1367*	*0.1199*	*0.1367*	*0.1801*	*0.1947*
Thanatophilus rugosus	*0.1276*	*0.1419*	*0.1293*	*0.1175*	*0.1334*	*0.1335*	*0.1310*	*0.1227*	*0.1179*	*0.1232*	*0.1298*	*0.1233*	*0.1189*	*0.1367*	*0.0000*	*0.0942*	*0.1135*	*0.1720*	*0.1881*
Thanatophilus sinuatus	*0.1094*	*0.1231*	*0.1082*	*0.1081*	*0.1146*	*0.1158*	*0.1146*	*0.1182*	*0.1002*	*0.1070*	*0.1110*	*0.1256*	*0.1134*	*0.1199*	*0.0942*	*0.0000*	*0.0805*	*0.1766*	*0.1833*
Thanatophilus capensis	*0.1264*	*0.1321*	*0.1195*	*0.1255*	*0.1319*	*0.1261*	*0.1283*	*0.1200*	*0.0997*	*0.1114*	*0.1227*	*0.1299*	*0.1188*	*0.1367*	*0.1135*	*0.0805*	*0.0000*	*0.1832*	*0.1968*
Aleochara curtula	*0.1921*	*0.1922*	*0.1713*	*0.1669*	*0.1706*	*0.1710*	*0.1734*	*0.1698*	*0.1534*	*0.1653*	*0.1708*	*0.1739*	*0.1722*	*0.1801*	*0.1720*	*0.1766*	*0.1832*	*0.0000*	*0.1957*
Scaphidium quadrimaculatum	*0.2031*	*0.2100*	*0.1865*	*0.1831*	*0.1983*	*0.1988*	*0.2000*	*0.1949*	*0.1836*	*0.1933*	*0.1915*	*0.2019*	*0.2012*	*0.1947*	*0.1881*	*0.1833*	*0.1968*	*0.1957*	*0.0000*

SM2: GenBank accession numbers of sequences used in this study. The newly generated sequences are marked with asterisk (*).

Species		COI	16S
Ingroup	*Ablattaria laevigata*	MW624526*	MW642419*
	Heterotemna tenuicornis Adult	MW633519*	MW642415*
	Heterotemna tenuicornis Larva (1)	MW633520 *	MW642416*
	Heterotemna tenuicornis Larva (2)	MW633521*	MW642417*
	Necrodes littoralis	AB606438.1	AB285536.1
	Necrodes nigricornis	AB606433.1	AB285544.1
	Oiceoptoma nigropunctatum	AB606470.1	AB285540.1
	Oiceoptoma subrufum	AB606486.1	AB285537.1
	Phosphuga atrata	MW624525*	MW642418*
	Silpha carinata	MW622082*	MW642412*
	Silpha obscura obscura	MW624523*	MW642413*
	Silpha olivieri	MW624524*	MW642414*
	Silpha perforata	AB438997.1	AB285534.1
	Silpha tristis	MW633518*	AB285542.1
	Thanatophilus mutilatus	MW700851*	MW680885*
	Thanatophilus rugosus	DQ155789.1	AB285546.1
	Thanatophilus sinuatus	JN086498.1	AB285548.1
Outgroup	*Aleochara curtula*	JN086504.1	JX536396.1
	Scaphidium quadrimaculatum	DQ221993.1	DQ202582.1

SM3: Collection information regarding specimens of *H. tenuicornis* used in this study.

Species	Developmental stage	Locality	Date
Heterotemna tenuicornis	Larva	SPAIN, Canary Islands: Tenerife, Anaga Mts., El Bailadero env., 28°32'55.8"N 16°11'12.3"W	22-27.XII. 2007
Heterotemna tenuicornis	Larva	SPAIN, Canary Islands: Tenerife, Anaga Mts., Chamorga env., ca. 28°34'30.5"N 16°09'37.0"W	16.II. 2011
Heterotemna tenuicornis	Larva	SPAIN, Canary Islands: Tenerife, Anaga Mts., [Cruz de Taborno], 28.53665°N 16.27043°W	19–20.XII. 2017
Heterotemna tenuicornis	Larva	SPAIN, Canary Islands: Tenerife, Anaga Mts., [Cruz de Taborno], 28.53665°N 16.27043°W	19–20.XII. 2017
Heterotemna tenuicornis	Adult	SPAIN, Canary Islands: Tenerife, Anaga	1.III.2010

SM4: Measurements and ratios of all three larval stages of *H. tenuicornis* (in millimetres).

Specimen ID	Instar	max_palp_I_mm	max_palp_II_mm	max_palp_III_mm	Antennomere_I_mm	Antennomere_II_mm	Antennomere_III_mm	Ratio_Antennomere I_II	Ratio_Anntenomere_II_III	Ratio_Anntenomere_I_III	Seta_Urog_mm	Urog_Seg_I_mm	Urog_Seg_II_mm	Ratio_Urogomphy_II_I	Ratio_Urogomphy_I_II	Head_Width_mm	Pronotum_Width_mm	Body_mm
H1	L1	0,17	0,22	0,38	0,72	0,7	0,85	1,03	0,82	0,85	0,3	0,61	0,21	0,34	0,34	1,93	5,66	
H10	L1	0,15	0,15	0,39	0,64	0,67	0,84	0,96	0,8	0,76	0,29	0,53	0,19	0,36	0,36	1,93	5,83	
H14	L1	0,16	0,18	0,28	0,74	0,71	0,87	1,04	0,82	0,85	0,32	0,64	0,24	0,38	0,38	1,87	5,33	
H16	L1	0,15	0,19	0,38	0,68	0,73	0,67	0,93	1,09	1,01	0,33	0,63	0,21	0,33	0,33	1,92	5,24	
H17	L1	0,16	0,19	0,3	0,68	0,67	0,84	1,01	0,8	0,81	0,28	0,59	0,24	0,41	0,41	1,94	5,7	
H18	L1	0,17	0,23	0,43	0,71	0,74	0,84	0,96	0,88	0,85	0,3	0,55	0,22	0,4	0,4	1,89	5,64	
H19	L1	0,16	0,19	0,31	0,83	0,77	0,91	1,08	0,85	0,91	0,34	0,67	0,19	0,28	0,28	1,99	5,69	
H2	L1	0,15	0,21	0,45	0,78	0,75	0,87	1,04	0,86	0,9	0,25	0,55	0,2	0,36	0,36	1,87	5,62	
H20	L1	0,21	0,25	0,24	0,68	0,7	0,78	0,97	0,9	0,87	NA	0,65	0,2	0,31	0,31	1,98	6,09	
H21	L1	0,15	0,21	0,41	0,71	0,71	0,83	1	0,86	0,86	0,32	0,62	0,21	0,34	0,34	1,89	5,4	
H22	L1	0,13	0,21	0,42	0,69	0,74	0,87	0,93	0,85	0,79	0,34	0,77	0,24	0,31	0,31	1,97	5,74	
H23	L1	0,13	0,2	0,4	0,72	0,71	0,87	1,01	0,82	0,83	0,33	0,66	0,25	0,38	0,38	1,91	5,74	
H24	L1	0,12	0,17	0,39	0,88	0,73	0,89	1,21	0,82	0,99	0,27	0,68	0,21	0,31	0,31	1,96	5,35	
H25	L1	0,13	0,2	0,37	0,72	0,66	0,76	1,09	0,87	0,95	0,25	0,59	0,18	0,31	0,31	1,94	5,58	
H26	L1	0,19	0,21	0,46	0,79	0,75	0,84	1,05	0,89	0,94	0,34	0,56	0,19	0,34	0,34	2	5,84	
H27	L1	0,34	0,27	0,17	0,57	0,65	0,77	0,88	0,84	0,74	0,28	0,47	0,22	0,47	0,47	1,96	5,75	
H28	L1	0,15	0,18	0,44	0,72	0,68	0,85	1,06	0,8	0,85	0,36	0,67	0,23	0,34	0,34	1,89	5,83	
H29	L1	0,11	0,23	0,4	0,7	0,7	0,85	1	0,82	0,82	0,27	0,58	0,22	0,38	0,38	1,95	6,11	
H3	L1	0,14	0,2	0,35	0,84	0,71	0,84	1,18	0,85	1	0,28	0,7	0,21	0,3	0,3	2,01	6,19	
H30	L1	0,14	0,17	0,41	0,68	0,62	0,85	1,1	0,73	0,8	0,3	0,55	0,16	0,29	0,29	1,93	5,36	
H31	L1	0,16	0,22	0,49	0,81	0,73	0,92	1,11	0,79	0,88	0,27	0,72	0,2	0,28	0,28	1,98	5,78	
H32	L1	0,16	0,2	0,38	0,6	0,64	0,77	0,94	0,83	0,78	0,3	0,59	0,18	0,31	0,31	1,89	5,89	
H33	L1	0,14	0,2	0,4	0,61	0,72	0,83	0,85	0,87	0,73	0,28	0,65	0,23	0,35	0,35	1,83	5,3	
H39	L1	0,14	0,16	0,42	0,63	0,61	0,76	1,03	0,8	0,83	NA	0,53	0,26	0,49	0,49	1,94	5,76	
H40	L1	0,14	0,23	0,41	0,61	0,66	0,68	0,92	0,97	0,9	NA	0,62	0,19	0,31	0,31	1,89	5,91	
H41	L1	0,15	0,2	0,42	0,81	0,77	0,83	1,05	0,93	0,98	NA	0,63	0,22	0,35	0,35	1,96	5,93	
H42	L1	0,09	0,19	0,41	0,53	0,71	0,86	0,75	0,83	0,62	NA	0,64	0,27	0,42	0,42	1,97	5,9	11,7
H43	L1	0,14	0,16	0,33	0,76	0,74	0,81	1,03	0,91	0,94	NA	0,54	0,17	0,31	0,31	1,92	5,85	13,53
H7	L1	0,18	0,22	0,4	0,72	0,67	0,74	1,07	0,91	0,97	0,21	0,6	0,14	0,23	0,23	1,85	5,66	
H9	L1	0,21	0,14	0,45	0,82	0,73	0,8	1,12	0,91	1,03	0,16	0,65	0,18	0,28	0,28	1,85	5,72	
H12	L2	0,2	0,22	0,27	0,88	0,87	1,01	1,01	0,86	0,87	0,15	0,83	0,18	0,22	0,22	2,43	6,38	
H13	L2	0,24	0,25	0,22	1,3	0,92	1,01	1,41	0,91	1,29	0,16	0,75	0,24	0,32	0,32	2,19	6,53	
H15	L2	0,19	0,24	0,42	0,89	0,95	0,87	0,94	1,09	1,02	0,14	0,86	0,15	0,17	0,17	2,19	6,46	13,34
H34	L2	0,21	0,22	0,22	0,93	0,92	1,11	1,01	0,83	0,84	0,17	0,81	0,18	0,22	0,22	2,27	6,79	
H35	L2	0,15	0,27	0,39	1,04	0,92	1	1,13	0,92	1,04	0,17	0,76	0,23	0,3	0,3	2,3	7,09	
H36	L2	0,2	0,27	0,45	0,87	0,9	1,02	0,97	0,88	0,85	0,17	0,81	0,13	0,16	0,16	2,43	7,34	
H37	L2	0,2	0,27	0,45	0,91	0,86	1,02	1,06	0,84	0,89	0,14	0,86	0,15	0,17	0,17	2,33	7,47	
H38	L2	0,2	0,27	0,45	0,91	0,85	1,03	1,07	0,83	0,88	0,17	0,8	0,17	0,21	0,21	2,37	7,08	
H45	L2	0,13	0,26	0,42	0,98	0,96	0,92	1,02	1,04	1,07	0,11	0,86	0,19	0,22	0,22	2,37	7,06	13,28
H46	L2	0,15	0,26	0,47	1,07	0,88	0,82	1,22	1,07	1,3	0,13	0,82	0,18	0,22	0,22	2,34	7,12	
H47	L2	0,17	0,2	0,45	0,92	0,95	0,94	0,97	1,01	0,98	NA	0,68	0,19	0,28	0,28	2,33	6,91	13,53
H5	L2	0,21	0,27	0,44	1,06	0,9	0,79	1,18	1,14	1,34	0,12	0,75	0,18	0,24	0,24	2,38	6,77	15,4
H6	L2	0,15	0,24	0,47	1,18	0,92	0,99	1,28	0,93	1,19	0,16	0,8	0,23	0,29	0,29	2,39	6,45	
H8	L2	0,23	0,21	0,43	1,04	0,88	0,94	1,18	0,94	1,11	0,18	0,87	0,23	0,26	0,26	2,26	7,26	15,97
H11	L3	0,26	0,21	0,43	1,35	1,19	1,25	1,13	0,95	1,08	0,13	0,94	0,21	0,22	0,22	2,84	8,58	15,83
H4	L3	0,24	0,32	0,5	1,39	0,92	1,19	1,51	0,77	1,17	0,16	1,01	0,26	0,26	0,26	2,91	8,45	
H44	L3	0,25	0,3	0,47	1,39	1,12	1,23	1,24	0,91	1,13	NA	0,96	0,23	0,24	0,24	2,72	8,39	
H48	L3	NA	NA	NA	1,51	1,28	1,2	1,18	1,07	1,26	0,14	1,11	0,23	0,21	0,21	2,91	9,11	17,46
